# Her Health, Common Dangers and Salvation

**Published:** 1918-08-17

**Authors:** W. J. O'Donovan

**Affiliations:** Chief Medical Officer to the Ministry of Munitions.


### THE HEALTH OF THE YOUNG GIRL IN INDUSTRY.

**By Dr. W. J. O'DONOVAN, M.R.C.P., Chief Medical Officer to the Ministry of Munitions.**

Preventive medicine may find its field of work in improving the environment of the population whether in their homes or in their workshops, or it may direct its efforts to so preserving the life of young people that the occurrence of later disability, or even fatal illnesses, may be prevented. Under the former comes the provision of good sanitary arrangements, condemnation of unwholesome food, the segregation of acute infectious illnesses, and the maintenance of a pure-water supply. These activities represent the main theme dealt with in the text-books on public health.

Another aspect from which the environment of the people can be improved lies in alleviating the conditions of industrial life. Great attention has been given in this regard towards the prevention of specified industrial diseases, such as lead poisoning, anthrax, and to-day poisoning by T.N.T. The activities that I have just recited, however, are self-evident fields of work. The possibility of a typhoid epidemic calls for a pure-water supply, the occurrence of palsied hands or unbearable colic pains calls for the prevention of lead poisoning, but the constant presence of a heavy load of sickness in our midst is accepted as an unalterable decree of Providence, and much that is preventable is not prevented.

The working girl, once she enters into factory life, benefits little from maternal control. Her occupation is settled by the persuasion of her friends or the attraction of a wage; as to her physical fitness for the work she undertakes, and its effect on her health—this is nobody's business. An attempt is made by the State to remedy this by insisting that young persons should be examined and passed by the certifying surgeon at the factory, but this examination is valued by the State at 6d., and is not repeated once the girl has been taken on for employment, however much the employment may have changed and whatever effect, good or bad, it may have upon her. In her extreme youth she has been watched by the school medical officer, and yet the valuable records of this public service are not available for the information of the certifying surgeon. The infectious diseases of childhood are followed by many disabling complications which may need much time for their elucidation. The school medical officer, owing to his repeated inspections, would have all these accurately recorded. The factory surgeon has no time for this anaylsis of the worker's past, and hence the work of a factory surgeon is done in ignorance and in haste. The correlation of the work of these two officers without the suppression of either would be a great step forward in organising care of the health of both young boys and young girls.

### Mother and Welfare Superintendent.

When, however, the young girl has been passed into industry, we are then faced with the important problem of adapting, as far as can be, industrial life to the physique and recognised weaknesses of the adolescent girl. There is no question that the girl's first and main protector is her mother; she, better than anyone else, can watch the girl's spirits, her appetite, and her bodily functions. She can advise with authority as to proper clothes, proper diet, and as regards domestic medicine, but it is unhappily true that many mothers are careless, and others stupidly unaware of the preciousness of the health of their own offspring, and until definite illness has impaired the girl's earning capacity no attention is given to her increasing debility. The welfare superintendent in a factory, observant, educated, and humane, can do much to remedy the absence of maternal care, and with special knowledge can go even further in protecting young girls' health.

There is no mystery about functions that establish themselves in puberty, and the menace to health does not arise here; but at this time growth is rapid, and the seeds of illness may now find a lodging in any part of the frame and disable the girl not now, but years hence.

### Some Common Dangers.

The dangers of rheumatic fever and tonsilitis are recognised by few that have not had a medical or nursing training; young girls run about hatless and insufficiently clad or shod in all weathers, and an attack of sore throat or rheumatic fever may at any time lead to a severe temporary illness, and the patient afterwards may appear well. Our heart hospitals are full of women whose useful lives have ended for good and all about the age of thirty owing to valvular disease of the heart caused definitely by rheumatic fever when they were young girls. Rheumatic fever in youth, and a failing heart in the prime of life, are a cause and effect which, if more widely known, would be more generally guarded against.

### An Unromantic Disability.

Less dramatic is the disability known as flat feet. Many men and women must be known to all of us unfit for active work owing to the pain and clumsiness of their flat feet, yet this condition started in youth and was then preventable. When young, they complain of tiredness in their legs and aching in their feet, and an examination of the soles of their boots would have shown the unnatural posture their feet were assuming; yet, for want of care at the time, and the use of simple exercises, to-day tnese sufferers have to wear surgical boots, or undergo painful operations.

Swollen glands of the neck are common in young people, yet their parents may not bring them to hospital until abscesses have formed, and prolonged treatment and extensive operations on the neck may be needed. Yet had a clean head been insisted upon the gland might not have enlarged at all, and had treatment been sought when the swelling was first observable, the whole disease might have been dissolved and operation avoided.

Anæmia is so frequently the subject of patent medicine advertisements in the press that many do not treat this subject as seriously as it deserves. Yet between the ages of nineteen and twenty-five there are hundreds of girls completely unfitted for work by weakness, breathlessness, indigestion, and swollen ankles, and to cure them at this stage may take anything from three to six months. Had they been directed to their doctor at the beginning of this condition in their first years of industrial life, the grave state of anæmia would never have manifested itself.

### Importance of Early Treatment.

It is only too true that many of the workers will give three or four years of neglect to establish a disease, and then they will be dissatisfied if their medical practitioner does not cure them in three or four weeks of treatment. Every welfare superintendent is familiar with the young girl who goes about all day with an open mouth, a pinched nose, and a nasal voice, and most recognise

**The Health of the Young Girl in Industry**—*cont.*

this as a condition due to adenoids. Such workers should never be put on any dusty employment, or chronic chest trouble will certainly ensue.

Teeth and Hair Needs.

A dentist is a person that the young wage-earner will hardly ever consult, unless in actual pain, yet a great part of the minor illnesses of later life is due to diseased teeth, and to the imperfect mastication of food. I need lay no stress upon the disfigurement of carious teeth, and many a stupid little girl could be persuaded to visit the dentist if the ugliness of her mouth and the unpleasantness of her breath were pointed out to her. An unpleasant possession that is also a matter of medical importance is that of nits in the hair. Many do not know where to look for nits, and it should be more widely known that small sores on the head are produced by scratching, and that through these sores germs gain access to the glands of the neck, and so predispose them to later tuberculosis. Nits, if present, will always be found at the back of the head just behind the ears, where there is warmth and an infrequent application of the hairbrush, and every welfare superintendent should know how to advise a girl to cleanse her hair efficiently.

A Curable Deformity.

Orthopædic surgeons have frequently to order what they call "leather jackets," with steel supports, for women for permanent curvature of the spine. This curvature in later life is sufficiently painful to prevent activity, and the deformity itself often causes the sufferer to withdraw herself from much intercourse with her fellows. There was a time, however, when by exercise, massage, and fresh air this condition was curable. Observant mothers will have noticed that the girl's shoulder was growing out, and similarly an observant welfare superintendent, noticing a crooked back, will direct a girl to her doctor for remedial measures, but, in addition, she will see that this girl does not return to work needing a cramped posture that will aggravate her condition. Young girls have an intense dislike to spectacles, yet any girl with perpetually sore eyes, or who works with her head close down to the process, should be directed to a skilled oculist.

Work for the Expectant Mother.

The last subject on which I would speak briefly is that of the employment of pregnant women. Employment in this condition, when it is recognisable, is not harmful, but, on the other hand, owing to the regularity of life that it ensures, and the wages that mean good food, it is in itself beneficial. It is a common belief that hard work induces miscarriages, and acting on this belief employers commonly discharge women when their condition is evident, yet, when pregnancy is evident, the risk of miscarriage has passed. Miscarriage occurs in the earliest months, whereas pregnancy may not be noticeable until the sixth month, or even later. The true line of advance here is to let it be understood throughout a factory that the welfare superintendent desires to know the onset of pregnancy at the very earliest possible time, and that in return for this confidence, not dismissal, but the provision of quiet work will at once follow, though as to the actual work that can be done, the woman herself is often the better judge.

Neurosis and its Manifestations.

So far I have spoken of deformities and diseases that can be seen, of which the effects can be more or less correctly measured, and yet there is a field of illness entailing grave disability. There is little information given in medical text-books on this subject, and it is hardly at all taught to the medical student, and yet, as every practitioner knows, its effects are felt in every family in the land. I refer to the great medical problem of neurosis in any of its protean manifestations. In its minor forms it is recognised as a constant debility and chronic languor with irritability and a growing depression. Hysterical attacks are only peaks raised above the surface of a sea of nervous illness. Nerve crises and nerve storms are but occasional incidents in a long history of individual nervous illness. Such cases, when they come to the doctor, are firmly established with a history of many years' standing. They are most difficult to cure, and their prevention needs every care and study that can be given. In the first place, a girl of fourteen is suddenly plunged out of five hours a day pleasant schooling, interspersed with long holidays, into a twelve-hour day of industrial life, and instead of living and playing amongst the young, she is surrounded by women of all ages and temperaments of a varied experience of life, who, as a rule, pay no regard to the feelings and immaturity of the young girls around them. Suddenly the young girl is faced with the constant thought that bad work is penalised by "the sack," which means trouble at home as well as in the factory. The effect of a harsh and unsympathetic forewoman on the mental stability of a young girl is very harmful. The exhaustion produced by the constant suppression of all her natural emotions, day by day and week after week, with its natural results, needs no stress on my part, and almost every factory has the unhealthy feature of noise hammering away continually at both auditory nerves, producing an exhaustion by the very effort of suppressing this noise from the threshold of consciousness. It is in factory life, too, that young girls go through the same experience of highly emotional friendships with one another, that can be watched and guarded against in High Schools, but which among the poor and ignorant are ill understood and less sympathised with.

Symptoms for the "Welfare" Officer.

From what I have said, you will gather that most of the nervous states, which end in recognised nervous illnesses, are in fact due to a suppression of individuality which our schools and colleges prize and cherish, but which in factory life is considered out of place and a hindrance to output. Here a welfare supervisor has her chance to play a part in lessening the load of nervous illness in later life. Children whose parents are known as neurotics, children whose past is marked by St. Vitus' dance, should be directed away from noisy or speedy repetition work and put, if possible, on to individual employment. Young people with epilepsy should be sent to hospital for treatment before the disease is ineradicable. Welfare supervisors should be on the lookout for twitchings of the face, or fidgeting of the fingers, for the frequent frownings that mark out the nervous girl, and special efforts should be made to obtain the confidence of such a one, and any possible source of personal anxiety should be alleviated. Hysteria is not a causeless phenomenon. It represents the exhaustion of long-accumulated nervous irritation, and once the attack is over the welfare supervisor should find out, if possible, what fault in the home, in friendship, or in factory life has been the prime cause of this emotional outbreak.

A Suggestion on Hospital Support.

I have said in my foregoing remarks that "you should do this," that "you should do that," and it is only right that I should conclude by pointing out the way in which the special medical assistance or advice can be obtained. Workpeople are extraordinarily generous in their contributions to the local hospitals, but the welfare superintendent should interest herself in this matter, so that the factory contributions may be used to secure the maximum benefit to the subscribers. The total sum should not go to some favourite hospital that treats acute medical or surgical conditions only, but a certain amount should be diverted to every special hospital or institution within reach, so that the opinion of an oral specialist, or an ophthalmologist, a specialist in women's diseases, trusses, or convalescent facilities, may be within the reach of every worker.

